# (9*S*,13*R*,14*S*)-7,8-Didehydro-4-(4-iodo­benz­yloxy)-3,7-dimeth­oxy-17-methyl­morphinan-6-one monohydrate

**DOI:** 10.1107/S1600536810040286

**Published:** 2010-10-20

**Authors:** Xing-Liang Zheng, Ning-Fei Jiang

**Affiliations:** a School of Chemistry and Biological Engineering, Changsha University of Science & Technology, Changsha 410114, People’s Republic of China

## Abstract

In the title compound, C_26_H_28_INO_4_·H_2_O, benzene rings are inclined at a dihedral angle of 69.9 (1)°. The N-containing ring exhibits a chair conformation, while the other rings approximate to envelope conformations. In the crystal, the uncoordinated water mol­ecule forms inter­molecular O—H⋯O and O—H⋯N hydrogen bonds.

## Related literature

For the biological activity of sinomenine derivatives and other related compounds, see: Liu *et al.* (1994 ▶, 1996 ▶, 1997 ▶); Mark *et al.* (2003 ▶); Ye *et al.* (2004 ▶). For the synthesis of the title compound, see: Mitsunobu (1981 ▶). For related structures, see: Li *et al.* (2009 ▶); Batterham *et al.* (1965 ▶).
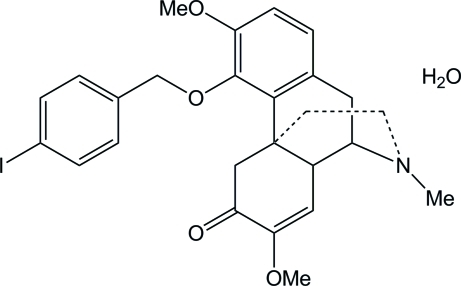


## Experimental

### 

#### Crystal data

C_26_H_28_INO_4_·H_2_O
*M* *_r_* = 563.41
Monoclinic, 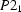

*a* = 8.9005 (8) Å
*b* = 14.9221 (14) Å
*c* = 9.2426 (9) Å
β = 91.432 (2)°
*V* = 1227.2 (2) Å^3^
*Z* = 2
Mo *K*α radiation
μ = 1.34 mm^−1^
*T* = 293 K
0.31 × 0.30 × 0.23 mm

#### Data collection

Bruker SMART APEX CCD area-detector diffractometer
Absorption correction: multi-scan (*SADABS*; Bruker, 2000 ▶) *T*_min_ = 0.482, *T*_max_ = 1.000
7231 measured reflections
5047 independent reflections
4844 reflections with *I* > 2σ(*I*)
*R*_int_ = 0.038

#### Refinement

*R*[*F* ^2^ > 2σ(*F* ^2^)] = 0.036
*wR*(*F* ^2^) = 0.087
*S* = 1.07
5047 reflections
309 parameters
4 restraints
H atoms treated by a mixture of independent and constrained refinement
Δρ_max_ = 0.98 e Å^−3^
Δρ_min_ = −0.49 e Å^−3^
Absolute structure: Flack (1983 ▶), 2311 Friedel pairs
Flack parameter: −0.016 (17)

### 

Data collection: *SMART* (Bruker, 2000 ▶); cell refinement: *SAINT* (Bruker, 2000 ▶); data reduction: *SAINT*; program(s) used to solve structure: *SHELXS97* (Sheldrick, 2008 ▶); program(s) used to refine structure: *SHELXL97* (Sheldrick, 2008 ▶); molecular graphics: *SHELXTL* (Sheldrick, 2008 ▶); software used to prepare material for publication: *SHELXTL*.

## Supplementary Material

Crystal structure: contains datablocks global, I. DOI: 10.1107/S1600536810040286/bh2309sup1.cif

Structure factors: contains datablocks I. DOI: 10.1107/S1600536810040286/bh2309Isup2.hkl

Additional supplementary materials:  crystallographic information; 3D view; checkCIF report

## Figures and Tables

**Table 1:** Hydrogen-bond geometry (Å, °)

*D*—H⋯*A*	*D*—H	H⋯*A*	*D*⋯*A*	*D*—H⋯*A*
O5—H5*C*⋯O3^i^	0.84 (3)	2.13 (3)	2.946 (5)	164 (6)
O5—H5*D*⋯N1^ii^	0.84 (3)	2.26 (11)	2.924 (6)	135 (13)

Symmetry codes: (i) 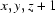; (ii) 
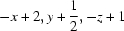
.

**Fig. 1.**
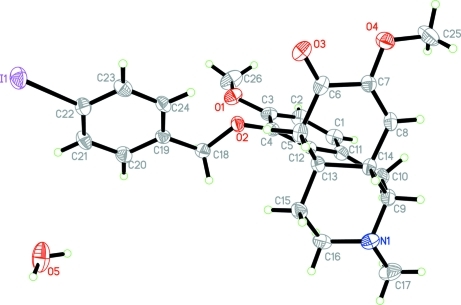
The molecular structure of (I) showing 50% probability displacement ellipsoids.

**Fig. 2.**
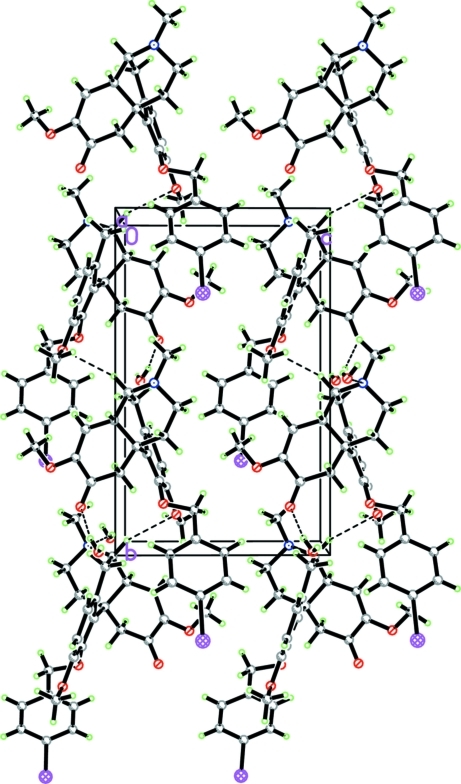
Packing structure of the title compound.

## supplementary crystallographic information

### Comment

We synthesized a new sinomenine derivative (9*S*,13*R*,14*S*)-7,8-didehydro-4-(4'-iodiobenzyloxy)-3,7-dimethoxy-17-methyl-morphinan-6-one monohydrate. Herein, its crystal structure is reported. Biological effects of sinomenine derivatives and related compounds have been described (Liu *et al.*, 1994, 1996, 1997; Mark *et al.*, 2003; Ye *et al.*, 2004).

The molecular structure of (I) is shown in Fig. 1. The crystal structure is stabilized by O—H···O and O—H···N hydrogen bonds linking sinomenine derivative and the water molecule, and weak C—H···O hydrogen bonds between molecules (Fig. 2). Significant aromatic stacking interactions were not found. There exist two planes in the molecule of the title compound: atoms C1/C2/C3/C4/C12/C11 form the benzene plane (I), and atoms C19···C24 form the benzene plane substituted by Iodine (II). The angle between the two planes is 69.9 (1)°. Rings *C* [C5/C6/C7/C8/C14/C13] and *B* [C9···C14] in the molecule approximate both an envelope conformation. In contrast, ring *D* [C9/N1/C16/C15/C13/C14] exhibits an almost regular chair conformation. Similar features have been described in related compounds (Li *et al.*, 2009; Batterham *et al.*, 1965).

### Experimental

The title compound was obtained according to the method of Mitsunobu (1981). Light yellow blocks of (I) were grown from a dichloromethane solution.

### Refinement

The water H atoms (H5C and H5D) were located in a difference map, and refined freely, although the geometry was restrained to O—H = 0.83 (3) Å and H5C···H5D separation to 1.45 (2) Å. Other H atoms were positioned geometrically, with C—H = 0.93 (aromatic CH), 0.96 (methyl CH_3_), 0.97 (methylene CH_2_) or 0.98 Å (methine CH), and were constrained to ride on their parent atoms, with *U*_iso_(H) = 1.2*U*_eq_(carrier C) or *U*_iso_(H) = 1.5*U*_eq_(carrier C). 2311 Friedel pairs were used for the Flack parameter refinement.

### Crystal data

**Table:**

C_26_H_28_INO_4_·H_2_O	*F*(000) = 572
*M**_r_* = 563.41	*D*_x_ = 1.525 Mg m^−^^3^
Monoclinic, *P*2_1_	Melting point: 412 K
Hall symbol: P 2yb	Mo *K*α radiation, λ = 0.71073 Å
*a* = 8.9005 (8) Å	Cell parameters from 4522 reflections
*b* = 14.9221 (14) Å	θ = 4.6–56.4°
*c* = 9.2426 (9) Å	µ = 1.34 mm^−^^1^
β = 91.432 (2)°	*T* = 293 K
*V* = 1227.2 (2) Å^3^	Prismatic, colourless
*Z* = 2	0.31 × 0.30 × 0.23 mm

### Data collection

**Table:**

Bruker SMART APEX CCD area-detector diffractometer	5047 independent reflections
Radiation source: fine-focus sealed tube	4844 reflections with *I* > 2σ(*I*)
graphite	*R*_int_ = 0.038
φ and ω scans	θ_max_ = 27.0°, θ_min_ = 2.2°
Absorption correction: multi-scan (*SADABS*; Bruker, 2000	*h* = −6→11
*T*_min_ = 0.482, *T*_max_ = 1.000	*k* = −18→19
7231 measured reflections	*l* = −11→11

### Refinement

**Table:**

Refinement on *F*^2^	Secondary atom site location: difference Fourier map
Least-squares matrix: full	Hydrogen site location: inferred from neighbouring sites
*R*[*F*^2^ > 2σ(*F*^2^)] = 0.036	H atoms treated by a mixture of independent and constrained refinement
*wR*(*F*^2^) = 0.087	*w* = 1/[σ^2^(*F*_o_^2^) + (0.0485*P*)^2^ + 0.0909*P*] where *P* = (*F*_o_^2^ + 2*F*_c_^2^)/3
*S* = 1.07	(Δ/σ)_max_ = 0.004
5047 reflections	Δρ_max_ = 0.98 e Å^−^^3^
309 parameters	Δρ_min_ = −0.49 e Å^−^^3^
4 restraints	Absolute structure: Flack (1983), 2311 Friedel pairs
0 constraints	Flack parameter: −0.016 (17)
Primary atom site location: structure-invariant direct methods	

### Fractional atomic coordinates and isotropic or equivalent isotropic displacement parameters (Å^2^)

**Table:**

	*x*	*y*	*z*	*U*_iso_*/*U*_eq_	
I1	1.01684 (3)	1.24508 (2)	0.40697 (3)	0.05793 (10)	
N1	0.7186 (4)	0.5021 (2)	0.1708 (4)	0.0481 (8)	
O1	0.3354 (3)	0.90402 (19)	0.2691 (3)	0.0528 (7)	
O2	0.6158 (3)	0.86212 (17)	0.2123 (2)	0.0381 (5)	
O3	0.7304 (3)	0.86778 (19)	−0.1786 (3)	0.0532 (7)	
O4	0.5676 (3)	0.7548 (3)	−0.3349 (3)	0.0504 (7)	
O5	0.9701 (6)	0.9909 (3)	0.9213 (6)	0.0898 (14)	
C1	0.2856 (4)	0.6717 (3)	0.1460 (5)	0.0474 (9)	
H1	0.2104	0.6290	0.1330	0.057*	
C2	0.2469 (4)	0.7561 (4)	0.1937 (4)	0.0497 (10)	
H2	0.1469	0.7705	0.2092	0.060*	
C3	0.3580 (4)	0.8183 (3)	0.2179 (4)	0.0412 (8)	
C4	0.5086 (4)	0.7971 (2)	0.1890 (4)	0.0342 (7)	
C5	0.7896 (4)	0.7650 (2)	0.0106 (4)	0.0386 (8)	
H5A	0.7968	0.8164	0.0745	0.046*	
H5B	0.8910	0.7451	−0.0082	0.046*	
C6	0.7159 (4)	0.7938 (2)	−0.1298 (4)	0.0392 (8)	
C7	0.6302 (4)	0.7238 (2)	−0.2098 (4)	0.0381 (8)	
C8	0.6280 (4)	0.6389 (2)	−0.1618 (4)	0.0407 (8)	
H8	0.5790	0.5955	−0.2178	0.049*	
C9	0.6273 (4)	0.5306 (2)	0.0456 (4)	0.0422 (8)	
H10	0.6290	0.4820	−0.0256	0.051*	
C10	0.4626 (4)	0.5535 (3)	0.0708 (4)	0.0471 (9)	
H11A	0.4055	0.5417	−0.0179	0.056*	
H11B	0.4254	0.5133	0.1443	0.056*	
C11	0.4318 (4)	0.6484 (3)	0.1170 (4)	0.0382 (7)	
C12	0.5457 (4)	0.7138 (2)	0.1318 (4)	0.0314 (7)	
C13	0.7065 (4)	0.6896 (2)	0.0881 (4)	0.0348 (7)	
C14	0.7012 (4)	0.6117 (2)	−0.0221 (4)	0.0364 (7)	
H9	0.8053	0.5952	−0.0422	0.044*	
C15	0.7966 (4)	0.6576 (3)	0.2203 (4)	0.0442 (8)	
H15A	0.8999	0.6473	0.1937	0.053*	
H15B	0.7967	0.7041	0.2938	0.053*	
C16	0.7328 (5)	0.5722 (3)	0.2826 (4)	0.0494 (9)	
H16A	0.7982	0.5511	0.3610	0.059*	
H16B	0.6349	0.5844	0.3218	0.059*	
C17	0.6656 (6)	0.4173 (4)	0.2330 (6)	0.0710 (14)	
H17A	0.5684	0.4262	0.2734	0.107*	
H17B	0.7352	0.3979	0.3077	0.107*	
H17C	0.6588	0.3725	0.1586	0.107*	
C18	0.6493 (5)	0.8804 (3)	0.3618 (4)	0.0435 (8)	
H18A	0.7019	0.8299	0.4057	0.052*	
H18B	0.5571	0.8900	0.4132	0.052*	
C19	0.7461 (4)	0.9628 (2)	0.3707 (4)	0.0369 (7)	
C20	0.7795 (5)	0.9999 (3)	0.5060 (4)	0.0464 (9)	
H20	0.7478	0.9713	0.5892	0.056*	
C21	0.8598 (5)	1.0793 (3)	0.5171 (4)	0.0479 (9)	
H21	0.8808	1.1045	0.6074	0.057*	
C22	0.9082 (4)	1.1204 (3)	0.3940 (4)	0.0410 (8)	
C23	0.8819 (4)	1.0833 (3)	0.2597 (4)	0.0443 (9)	
H23	0.9172	1.1110	0.1771	0.053*	
C24	0.8021 (4)	1.0042 (3)	0.2494 (4)	0.0398 (8)	
H24	0.7855	0.9781	0.1590	0.048*	
C25	0.4769 (6)	0.6931 (5)	−0.4172 (5)	0.0738 (16)	
H25A	0.5393	0.6463	−0.4539	0.111*	
H25B	0.4285	0.7241	−0.4966	0.111*	
H25C	0.4022	0.6677	−0.3564	0.111*	
C26	0.1839 (6)	0.9304 (4)	0.2891 (7)	0.0756 (16)	
H26A	0.1239	0.9135	0.2058	0.113*	
H26B	0.1794	0.9942	0.3016	0.113*	
H26C	0.1461	0.9014	0.3735	0.113*	
H5C	0.913 (6)	0.954 (4)	0.879 (7)	0.10 (2)*	
H5D	1.060 (5)	0.976 (7)	0.937 (14)	0.11 (2)*	

### Atomic displacement parameters (Å^2^)

**Table:**

	*U*^11^	*U*^22^	*U*^33^	*U*^12^	*U*^13^	*U*^23^
I1	0.05580 (15)	0.06209 (16)	0.05586 (15)	−0.02060 (14)	0.00072 (10)	−0.00469 (15)
N1	0.0504 (19)	0.0413 (17)	0.0528 (19)	0.0021 (14)	0.0037 (15)	0.0087 (14)
O1	0.0508 (16)	0.0474 (16)	0.0610 (17)	0.0130 (12)	0.0148 (13)	0.0009 (13)
O2	0.0438 (14)	0.0384 (13)	0.0321 (11)	−0.0044 (10)	0.0006 (10)	−0.0043 (10)
O3	0.0535 (17)	0.0367 (15)	0.0698 (18)	−0.0028 (12)	0.0062 (14)	0.0022 (13)
O4	0.0530 (14)	0.0547 (19)	0.0433 (12)	−0.0036 (16)	−0.0010 (10)	0.0055 (15)
O5	0.072 (3)	0.088 (3)	0.111 (3)	−0.028 (2)	0.029 (2)	−0.041 (3)
C1	0.0345 (18)	0.057 (3)	0.051 (2)	−0.0086 (17)	0.0049 (17)	0.0091 (18)
C2	0.0323 (15)	0.061 (3)	0.056 (2)	0.0032 (19)	0.0095 (14)	0.003 (2)
C3	0.0401 (19)	0.047 (2)	0.0368 (18)	0.0065 (16)	0.0071 (15)	0.0082 (15)
C4	0.0339 (18)	0.0390 (19)	0.0299 (17)	−0.0021 (14)	0.0027 (14)	0.0023 (14)
C5	0.0313 (15)	0.039 (2)	0.0459 (17)	−0.0061 (13)	0.0084 (13)	−0.0071 (13)
C6	0.0322 (17)	0.0351 (19)	0.051 (2)	−0.0019 (14)	0.0151 (15)	−0.0063 (15)
C7	0.0383 (16)	0.042 (2)	0.0345 (16)	−0.0025 (13)	0.0064 (13)	−0.0022 (13)
C8	0.046 (2)	0.0396 (19)	0.0372 (18)	−0.0082 (16)	0.0044 (15)	−0.0095 (15)
C9	0.049 (2)	0.039 (2)	0.0388 (18)	−0.0050 (16)	0.0037 (16)	−0.0026 (14)
C10	0.049 (2)	0.042 (2)	0.050 (2)	−0.0103 (16)	−0.0032 (17)	0.0006 (16)
C11	0.0362 (17)	0.0435 (19)	0.0349 (17)	−0.0070 (15)	−0.0007 (13)	0.0034 (14)
C12	0.0296 (16)	0.0366 (18)	0.0279 (16)	−0.0020 (12)	−0.0014 (13)	0.0027 (12)
C13	0.0327 (16)	0.0387 (18)	0.0330 (16)	−0.0015 (13)	0.0019 (13)	−0.0044 (14)
C14	0.0392 (19)	0.0327 (18)	0.0376 (17)	−0.0018 (14)	0.0065 (14)	−0.0045 (13)
C15	0.0372 (18)	0.052 (2)	0.044 (2)	0.0054 (16)	−0.0039 (15)	−0.0057 (16)
C16	0.047 (2)	0.059 (3)	0.042 (2)	0.0088 (18)	−0.0034 (17)	0.0075 (18)
C17	0.080 (4)	0.054 (3)	0.079 (3)	−0.002 (2)	0.002 (3)	0.024 (2)
C18	0.054 (2)	0.040 (2)	0.0365 (18)	0.0025 (17)	−0.0015 (16)	0.0011 (14)
C19	0.0350 (17)	0.0387 (18)	0.0369 (18)	0.0058 (14)	−0.0029 (14)	0.0005 (14)
C20	0.055 (2)	0.051 (2)	0.0335 (18)	−0.0047 (18)	0.0038 (16)	0.0015 (16)
C21	0.050 (2)	0.064 (3)	0.0301 (17)	−0.0084 (18)	0.0003 (15)	−0.0069 (16)
C22	0.0321 (17)	0.048 (2)	0.0431 (19)	−0.0019 (15)	0.0014 (14)	−0.0044 (15)
C23	0.0392 (19)	0.060 (2)	0.0335 (18)	−0.0021 (17)	0.0041 (14)	0.0028 (16)
C24	0.0414 (19)	0.049 (2)	0.0295 (16)	0.0028 (16)	−0.0013 (14)	−0.0054 (14)
C25	0.084 (4)	0.092 (4)	0.044 (3)	−0.024 (3)	−0.017 (3)	0.009 (2)
C26	0.063 (3)	0.065 (3)	0.100 (4)	0.023 (2)	0.036 (3)	0.007 (3)

### Geometric parameters (Å, °)

**Table:**

I1—C22	2.099 (4)	C10—H11B	0.9700
N1—C9	1.461 (5)	C11—C12	1.411 (5)
N1—C17	1.472 (6)	C12—C13	1.541 (5)
N1—C16	1.474 (6)	C13—C15	1.521 (5)
O1—C3	1.380 (5)	C13—C14	1.546 (4)
O1—C26	1.421 (5)	C14—H9	0.9800
O2—C4	1.374 (4)	C15—C16	1.516 (6)
O2—C18	1.432 (4)	C15—H15A	0.9700
O3—C6	1.201 (5)	C15—H15B	0.9700
O4—C7	1.353 (4)	C16—H16A	0.9700
O4—C25	1.430 (6)	C16—H16B	0.9700
O5—H5C	0.84 (3)	C17—H17A	0.9600
O5—H5D	0.84 (3)	C17—H17B	0.9600
C1—C11	1.380 (5)	C17—H17C	0.9600
C1—C2	1.381 (7)	C18—C19	1.502 (5)
C1—H1	0.9300	C18—H18A	0.9700
C2—C3	1.370 (6)	C18—H18B	0.9700
C2—H2	0.9300	C19—C24	1.384 (5)
C3—C4	1.410 (5)	C19—C20	1.393 (5)
C4—C12	1.392 (5)	C20—C21	1.387 (6)
C5—C6	1.501 (5)	C20—H20	0.9300
C5—C13	1.534 (5)	C21—C22	1.371 (5)
C5—H5A	0.9700	C21—H21	0.9300
C5—H5B	0.9700	C22—C23	1.375 (5)
C6—C7	1.480 (5)	C23—C24	1.379 (6)
C7—C8	1.343 (5)	C23—H23	0.9300
C8—C14	1.488 (5)	C24—H24	0.9300
C8—H8	0.9300	C25—H25A	0.9600
C9—C14	1.520 (5)	C25—H25B	0.9600
C9—C10	1.528 (5)	C25—H25C	0.9600
C9—H10	0.9800	C26—H26A	0.9600
C10—C11	1.507 (6)	C26—H26B	0.9600
C10—H11A	0.9700	C26—H26C	0.9600
			
C9—N1—C17	112.5 (4)	C8—C14—C13	111.8 (3)
C9—N1—C16	112.6 (3)	C9—C14—C13	109.5 (3)
C17—N1—C16	111.0 (4)	C8—C14—H9	107.4
C3—O1—C26	116.6 (4)	C9—C14—H9	107.4
C4—O2—C18	114.4 (3)	C13—C14—H9	107.4
C7—O4—C25	116.7 (4)	C16—C15—C13	111.9 (3)
H5C—O5—H5D	118 (5)	C16—C15—H15A	109.2
C11—C1—C2	122.4 (4)	C13—C15—H15A	109.2
C11—C1—H1	118.8	C16—C15—H15B	109.2
C2—C1—H1	118.8	C13—C15—H15B	109.2
C3—C2—C1	119.0 (3)	H15A—C15—H15B	107.9
C3—C2—H2	120.5	N1—C16—C15	110.9 (3)
C1—C2—H2	120.5	N1—C16—H16A	109.4
C2—C3—O1	124.9 (4)	C15—C16—H16A	109.4
C2—C3—C4	120.2 (4)	N1—C16—H16B	109.4
O1—C3—C4	114.9 (4)	C15—C16—H16B	109.4
O2—C4—C12	121.3 (3)	H16A—C16—H16B	108.0
O2—C4—C3	118.1 (3)	N1—C17—H17A	109.5
C12—C4—C3	120.5 (4)	N1—C17—H17B	109.5
C6—C5—C13	114.1 (3)	H17A—C17—H17B	109.5
C6—C5—H5A	108.7	N1—C17—H17C	109.5
C13—C5—H5A	108.7	H17A—C17—H17C	109.5
C6—C5—H5B	108.7	H17B—C17—H17C	109.5
C13—C5—H5B	108.7	O2—C18—C19	108.3 (3)
H5A—C5—H5B	107.6	O2—C18—H18A	110.0
O3—C6—C7	121.3 (4)	C19—C18—H18A	110.0
O3—C6—C5	122.6 (3)	O2—C18—H18B	110.0
C7—C6—C5	116.0 (3)	C19—C18—H18B	110.0
C8—C7—O4	126.6 (3)	H18A—C18—H18B	108.4
C8—C7—C6	120.8 (3)	C24—C19—C20	118.5 (4)
O4—C7—C6	112.5 (3)	C24—C19—C18	122.6 (3)
C7—C8—C14	122.3 (3)	C20—C19—C18	119.0 (3)
C7—C8—H8	118.8	C21—C20—C19	120.3 (4)
C14—C8—H8	118.8	C21—C20—H20	119.8
N1—C9—C14	108.7 (3)	C19—C20—H20	119.8
N1—C9—C10	117.5 (3)	C22—C21—C20	119.5 (4)
C14—C9—C10	108.2 (3)	C22—C21—H21	120.2
N1—C9—H10	107.4	C20—C21—H21	120.2
C14—C9—H10	107.4	C21—C22—C23	121.3 (4)
C10—C9—H10	107.4	C21—C22—I1	120.2 (3)
C11—C10—C9	115.8 (3)	C23—C22—I1	118.5 (3)
C11—C10—H11A	108.3	C22—C23—C24	118.9 (3)
C9—C10—H11A	108.3	C22—C23—H23	120.6
C11—C10—H11B	108.3	C24—C23—H23	120.6
C9—C10—H11B	108.3	C23—C24—C19	121.5 (3)
H11A—C10—H11B	107.4	C23—C24—H24	119.3
C1—C11—C12	119.0 (4)	C19—C24—H24	119.3
C1—C11—C10	118.1 (3)	O4—C25—H25A	109.5
C12—C11—C10	122.9 (3)	O4—C25—H25B	109.5
C4—C12—C11	118.5 (3)	H25A—C25—H25B	109.5
C4—C12—C13	122.6 (3)	O4—C25—H25C	109.5
C11—C12—C13	118.8 (3)	H25A—C25—H25C	109.5
C15—C13—C5	110.8 (3)	H25B—C25—H25C	109.5
C15—C13—C12	109.7 (3)	O1—C26—H26A	109.5
C5—C13—C12	114.3 (3)	O1—C26—H26B	109.5
C15—C13—C14	107.5 (3)	H26A—C26—H26B	109.5
C5—C13—C14	104.5 (3)	O1—C26—H26C	109.5
C12—C13—C14	109.6 (3)	H26A—C26—H26C	109.5
C8—C14—C9	112.9 (3)	H26B—C26—H26C	109.5
			
C11—C1—C2—C3	−2.2 (6)	C6—C5—C13—C12	−61.4 (4)
C1—C2—C3—O1	−178.2 (3)	C6—C5—C13—C14	58.5 (4)
C1—C2—C3—C4	2.4 (6)	C4—C12—C13—C15	84.0 (4)
C26—O1—C3—C2	−4.1 (6)	C11—C12—C13—C15	−94.3 (4)
C26—O1—C3—C4	175.4 (4)	C4—C12—C13—C5	−41.1 (4)
C18—O2—C4—C12	−110.7 (3)	C11—C12—C13—C5	140.6 (3)
C18—O2—C4—C3	72.3 (4)	C4—C12—C13—C14	−158.1 (3)
C2—C3—C4—O2	178.8 (3)	C11—C12—C13—C14	23.6 (4)
O1—C3—C4—O2	−0.7 (5)	C7—C8—C14—C9	152.3 (3)
C2—C3—C4—C12	1.9 (5)	C7—C8—C14—C13	28.2 (5)
O1—C3—C4—C12	−177.6 (3)	N1—C9—C14—C8	172.5 (3)
C13—C5—C6—O3	153.4 (3)	C10—C9—C14—C8	−59.0 (4)
C13—C5—C6—C7	−29.9 (4)	N1—C9—C14—C13	−62.2 (4)
C25—O4—C7—C8	6.9 (6)	C10—C9—C14—C13	66.4 (4)
C25—O4—C7—C6	−177.4 (4)	C15—C13—C14—C8	−174.5 (3)
O3—C6—C7—C8	172.9 (4)	C5—C13—C14—C8	−56.7 (4)
C5—C6—C7—C8	−3.9 (5)	C12—C13—C14—C8	66.3 (4)
O3—C6—C7—O4	−3.1 (5)	C15—C13—C14—C9	59.6 (4)
C5—C6—C7—O4	−179.9 (3)	C5—C13—C14—C9	177.3 (3)
O4—C7—C8—C14	179.8 (3)	C12—C13—C14—C9	−59.7 (4)
C6—C7—C8—C14	4.4 (5)	C5—C13—C15—C16	−169.2 (3)
C17—N1—C9—C14	−173.1 (4)	C12—C13—C15—C16	63.7 (4)
C16—N1—C9—C14	60.6 (4)	C14—C13—C15—C16	−55.5 (4)
C17—N1—C9—C10	63.8 (5)	C9—N1—C16—C15	−56.4 (4)
C16—N1—C9—C10	−62.6 (4)	C17—N1—C16—C15	176.4 (4)
N1—C9—C10—C11	86.5 (4)	C13—C15—C16—N1	54.0 (4)
C14—C9—C10—C11	−36.9 (4)	C4—O2—C18—C19	−170.1 (3)
C2—C1—C11—C12	−2.1 (6)	O2—C18—C19—C24	−6.3 (5)
C2—C1—C11—C10	177.7 (4)	O2—C18—C19—C20	172.8 (3)
C9—C10—C11—C1	−177.8 (3)	C24—C19—C20—C21	3.6 (6)
C9—C10—C11—C12	2.0 (5)	C18—C19—C20—C21	−175.6 (4)
O2—C4—C12—C11	177.0 (3)	C19—C20—C21—C22	−1.0 (6)
C3—C4—C12—C11	−6.2 (5)	C20—C21—C22—C23	−1.6 (6)
O2—C4—C12—C13	−1.3 (5)	C20—C21—C22—I1	175.7 (3)
C3—C4—C12—C13	175.5 (3)	C21—C22—C23—C24	1.5 (6)
C1—C11—C12—C4	6.3 (5)	I1—C22—C23—C24	−175.9 (3)
C10—C11—C12—C4	−173.6 (3)	C22—C23—C24—C19	1.2 (6)
C1—C11—C12—C13	−175.4 (3)	C20—C19—C24—C23	−3.7 (6)
C10—C11—C12—C13	4.8 (5)	C18—C19—C24—C23	175.4 (4)
C6—C5—C13—C15	174.0 (3)		

### Hydrogen-bond geometry (Å, °)

**Table:**

*D*—H···*A*	*D*—H	H···*A*	*D*···*A*	*D*—H···*A*
O5—H5C···O3^i^	0.84 (3)	2.13 (3)	2.946 (5)	164 (6)
O5—H5D···N1^ii^	0.84 (3)	2.26 (11)	2.924 (6)	135 (13)

Symmetry codes: (i) *x*, *y*, *z*+1; (ii) −*x*+2, *y*+1/2, −*z*+1.
